# Practical approach to prevent COVID-19 infection at breast cancer screening

**DOI:** 10.1007/s12282-021-01235-y

**Published:** 2021-04-02

**Authors:** Mamoru Takada, Gaku Tanaka, Hideyuki Hashimoto, Yasuyuki Hirai, Taichi Fukushima, Takeshi Nagashima, Masayuki Otsuka, Fumio Imazeki

**Affiliations:** 1 Safety and Health Organization, Chiba University, 1-33, Yayoi-cho, Inage-ku, Chiba-City, Chiba Japan; 2 Department of General Surgery, Graduate School of Medicine, Chiba University, Chiba, Japan; 3 Graduate School of Engineering, Chiba University, Chiba, Japan; 4 Breast Screening Center, Chiba Foundation for Health Promotion and Disease Prevention, Chiba, Japan

**Keywords:** COVID-19, Aerosol, Simulation, Ventilation, Breast cancer, Mobile Screening MMG bus services

## Abstract

**Background:**

The novel coronavirus disease 2019 (COVID-19) undermines the benefits of cancer screening. To date, no study has identified specific infection control methods. We aimed to provide practical methods for COVID-19 risk reduction during breast cancer screening mammography (MMG) by examining an overview of potential contamination routes of aerosols and possible risks for patients and health care providers.

**Methods:**

Computational fluid dynamics (CFD) simulations were conducted for airflow and aerosol dispersion in a 3D virtual model of a mobile MMG laboratory room. This model was constructed based on the actual mobile screening MMG bus ‘Cosmos’ in the Chiba Foundation for Health Promotion & Disease Prevention. Examiner and patient geometries were obtained by scanning an actual human using a 3D Scanner. Contamination of the room was evaluated by counting the numbers of suspended and deposited aerosols.

**Results:**

We applied the CFD simulation model to the exhalation of small or large aerosols from a patient and examiner in the MMG laboratory. Only 14.5% and 54.5% of large and small aerosols, respectively, were discharged out of the room with two doors open. In contrast, the proportion of large and small aerosols discharged out of the room increased to 96.6% and 97.9%, respectively, with the addition of forced gentle wind by the blower fan. This simulation was verified by a mist aerosol experiment conducted in the mobile MMG laboratory.

**Conclusion:**

Adding forced ventilation to a MMG laboratory with two doors open may enable risk reduction dramatically. This could be applied to other clinical situations.

**Supplementary Information:**

The online version contains supplementary material available at 10.1007/s12282-021-01235-y.

## Introduction

The 2019 novel human coronavirus named severe acute respiratory syndrome coronavirus 2 (SARS-CoV-2) or coronavirus disease 2019 (COVID-19) was first reported in December 2019 in Wuhan, China, and has developed into a pandemic [1]. SARS-CoV-2 is genetically close to SARS-CoV, which caused a global epidemic with 8096 confirmed cases in more than 25 countries in 2002–2003 [2]. SARS-CoV-2 is transmitted through human respiratory droplets, direct contact, and potentially aerosols, which drift through the air and accumulate over time; therefore, we can reduce the risk by social distancing [3, 4].

Transmission of respiratory infections of SARS-CoV-2 is associated with modest viral loads primarily via fluid particles (i.e., droplets and aerosols) that are formed in the respiratory tract during early illness, with viral loads peaking within several days after symptom onset in infected symptomatic patients as well as asymptomatic patients [5–8]. Additionally, less than one-third of patients infected with COVID-19 had a fever and/or respiratory symptoms, whereas 15–30% of patients were asymptomatic or displayed non-specific symptoms [9, 10]. SARS-CoV-2 aerosol distribution characteristics indicate that the transmission distance of SARS-CoV-2 might be 4 m [11]. However, in reality, it may not be feasible to provide protection during clinical practice, an essential activity, from virus-carrying aerosols released into the air from SARS-CoV-2 patients.

Aerosols are expelled from the mouth and nose during breathing, talking, coughing, and sneezing. Large aerosols settle faster than they evaporate and contaminate various surfaces with a viable virus titer [12]. Small aerosols evaporate faster than they settle, so they can float in the air for hours and be transported over long distances. Both aerosol sizes can accumulate in poorly ventilated venues and be carried in air currents. Human-to-human transmission of COVID-19 is thought to occur via two routes, which are (i) physical contact with droplets deposited on a surface and subsequent transfer to the recipient’s respiratory mucosa and (ii) the ‘airborne’ transmission route [13]. SARS-CoV-2 is spread by contaminated surfaces or through close contact. It has been elucidated that SARS-CoV-2 might spread through respiratory aerosols or droplets; to date, viral RNA has been identified from air sampling in some studies [11, 14].

The appropriate distance from a patient whose virus is infective is considered important; however, the optimum countermeasure during a clinical/medical checkup is not well studied.

Breast cancer screening with mammography (MMG) has been recommended for many years [12] and women older than 40 years old participate in screening activities, which reduce breast cancer mortality [14, 15]. Mobile Screening MMG bus services are provided to many parts of Japan and play an important role in increasing the overall rate of women being screened for breast cancer, detecting early stages of cancer and, therefore, improving prognosis in patients with breast cancer [16–18].

Due to fear of COVID-19 infection, people are hesitant to attend the clinical screening, so an appropriate method that can prevent airborne transmission and reduce infection risk, such as measures improving the ventilation of the indoor MMG laboratory, would be necessary to minimize the burden of illness on people worldwide by reducing the cancer morbidity and mortality rate. However, to date, such a method has not been well developed and there is limited supporting evidence. MMG, chest X-ray, or ultrasound for screening requires isolation from the surroundings to consider privacy, so the ventilation of these rooms is potentially challenging, especially in mobile service systems. Little has been reported on the simplification and effective achievement of ventilation in X-ray laboratories for removing aerosols as a risk management of COVID-19 or during post-COVID-19.

Here we report specific measures, on the basis of experimental data, for ventilation in clinical situations that can reduce the threat of emerging viral disease during and after the COVID-19 pandemic.

## Materials and methods

### Model

**Fig. 1:**
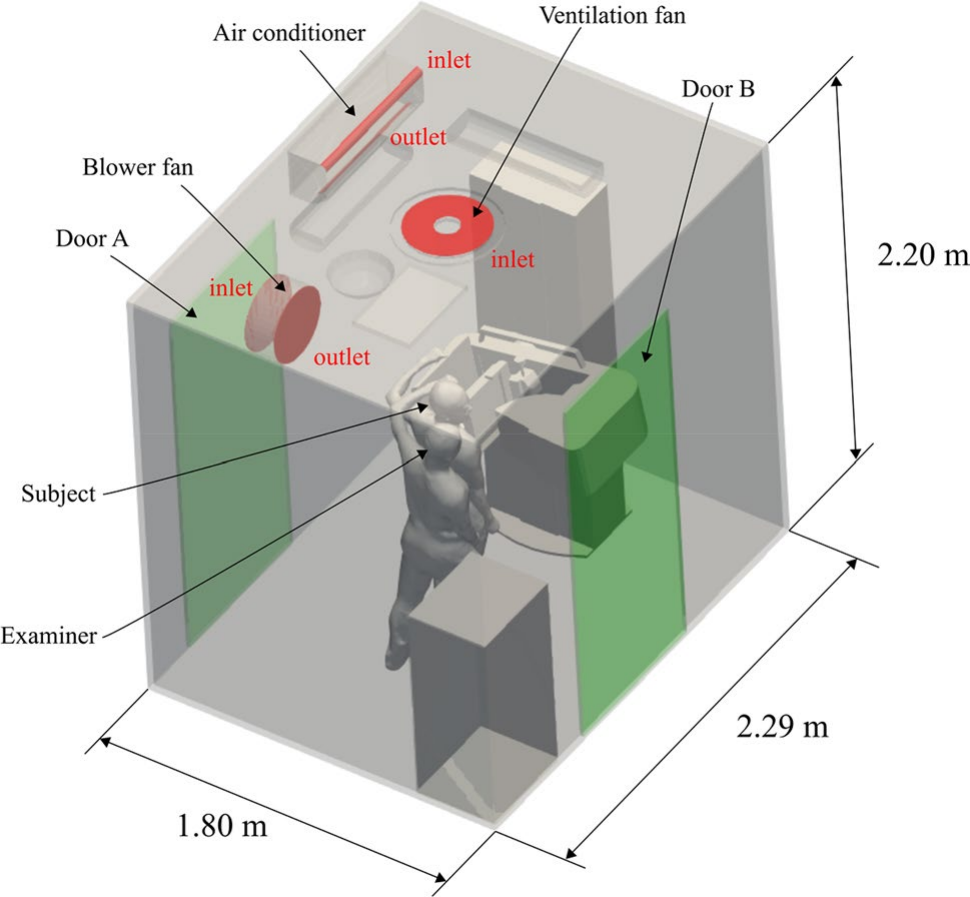
Model of a mobile MMG laboratory room

**Fig. 2:**
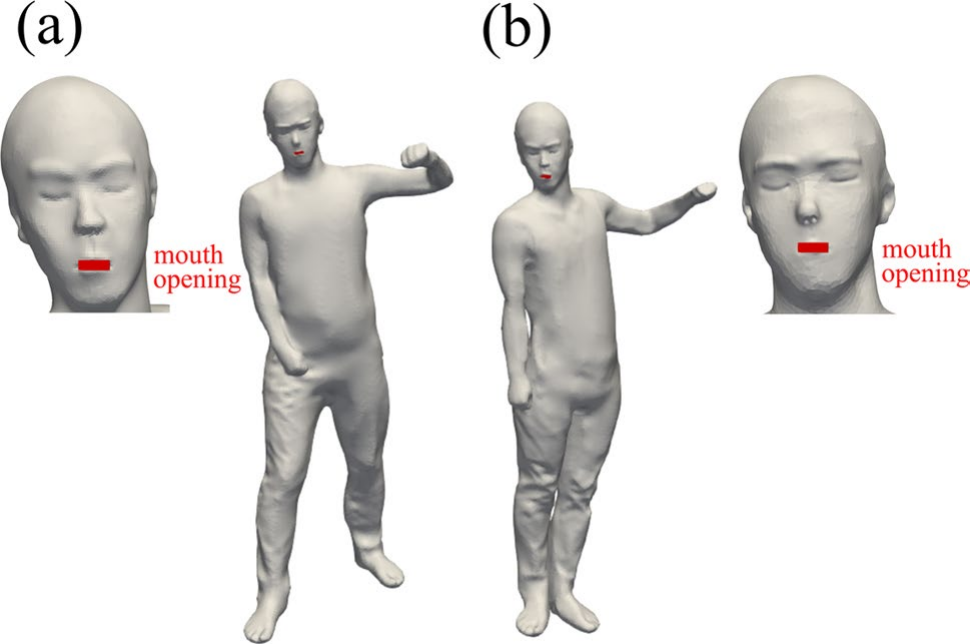
Geometrics of (**a**) examiner and (**b**) patient

We constructed a virtual model of a mobile MMG laboratory room using 3D CAD software (Inventor, Autodesk) based on the actual mobile screening MMG bus ‘Cosmos’ in the Chiba Foundation for Health Promotion & Disease Prevention (Fig. 1), and its structure is commonly used as MMG Screening vehicles in Japan. The contours, wind power, and wind direction at the inlets and outlets of the air-conditioning unit (red regions in Fig. 1) were measured to conduct a dispersion test for aerosols in the mobile MMG laboratory room by simulating a circulation ventilation situation in a room. Wind velocity was measured using a hot-wire anemometer (405i, testo). The geometries of the examiner and patient were obtained by scanning an actual human with a height of 175 cm using a 3D Scanner (Handheld 3D Scanner 2.0, XYZ Printing). Additionally, by reducing this 3D-scanned model in a self-similar manner, 155 cm (the average height of Japanese women) geometries of the examiner and patient (Fig. 2) were also created.

### Computational Fluid Dynamics (CFD) simulation

For simulating airflow in a mobile MMG laboratory room, the 3D unsteady Reynolds-averaged Navier–Stokes (RANS) equations were solved with the Shear Stress Transport (SST) k-ω turbulence model using the commercial CFD code STAR-CCM + Ver. 13.06 (Siemens PLM Software), with a second-order, segregated, flow solver based on the SIMPLE method [23]. The working fluid is air with constant density and viscosity under normal temperature and pressure (25 °C, 1 atm). Computational hybrid meshes of the laboratory room, combining interior polyhedral elements with a 5 layered prism mesh adjacent to the wall, were generated. The height of the minimum prism was 50 μm, and the total number of meshes was 1,468,068.

As a boundary condition, the constant exhaling velocity of 2.5 m/s was imposed uniformly at the mouth opening (Fig. 2) and was directed perpendicular to the opening plane. This velocity corresponds to moderately deep breathing. Similarly, the measured flow velocity of 2.4 m/s was imposed at the inlet of the ventilator in the ceiling (Fig. 1), and 1.6 m/s was imposed at the inlet and outlet of the air conditioner. In this study, simulations were performed with and without the blower fan near door A. Under the condition with the blower fan, the measured flow velocity of 2.5 m/s was imposed at the inlet and outlet of the ventilation fan. Doors A and B were open, and constant atmospheric pressure condition was imposed. A no-slip condition was applied to the other walls.

**Fig. 3:**
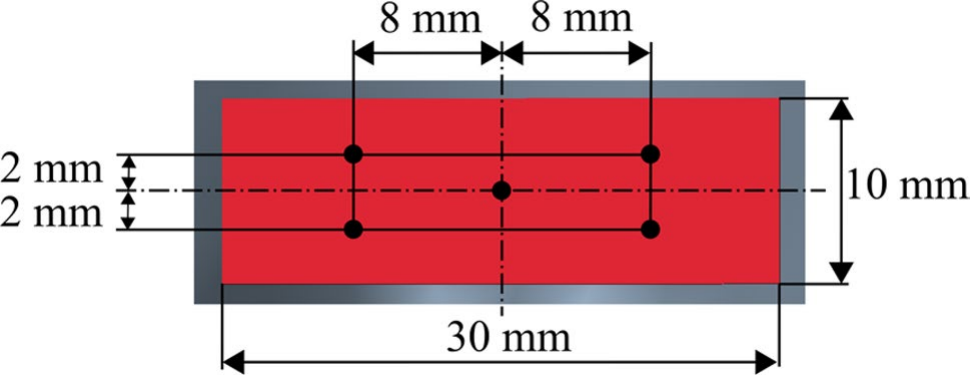
Position of particle emission on mouth opening

From the initial conditions (zero flow velocity and atmospheric pressure), the simulations of the airflow and particle tracking were performed for 15 s. The discrete phase model in the STAR-CCM + software was used to calculate particle tracks by integrating the particle velocity obtained from the equation of motion describing the particle motion in the Lagrange formulation. One-way coupling is assumed between the flow and particle equations. Particles were emitted from each of the 5 positions on the mouth opening (Fig. 3) at 1 particle per 1 time step (0.1 s) [19].

The particles were represented with a Rosin–Rammler distribution with a minimum diameter of 0.1 μm, average diameter of 10 (small particles) and 80 (large particles) μm, and a maximum diameter of 1000 μm. The particle density was set equal to that of water. The Stokes Number (= particle sedimentation velocity/exhaling velocity) calculated from the average diameter is 1.21 × 10^–2^ for small particles and 7.74 × 10^–2^ for large particles.

### Visualization experiment

To visualize the particle dispersion in the actual mobile MMG laboratory room, experiments were conducted using fine water mist and a continuous light YAG laser sheet (DPGL-2 W, JAPAN LASER). This was done by connecting an ultrasonic humidifier to the respiratory tract of the manikin. The exhaling velocity was adjusted to be the same as that in the CFD simulation (2.5 m/s). The mist images were recorded with a digital camera (iPhone XR, Apple). Five mist images were recorded at 1 s intervals within the 11.0 s to 15.0 s range, with the start of mist release as 0 s. (Fig. 5, Fig. 6) Mist images when the surgical non-woven mask was attached to the manikin were also acquired by the digital camera. (Suppl. Figure 3) In addition, only the mist regions of mist images were extracted using the region growing tool of an image editing software (Photoshop, Adobe). The areas of the extracted mist regions were then calculated using an image processing software (Fiji—Image J, National Institutes of Health).

## Results

For the deposition calculations, aerosol particles were characterized by a number size distribution with a median diameter of 10 nm as a small aerosol, 80 nm as a large aerosol, and a particle concentration of x × 10^9^ cm^−3^ [5, 20–22]. Measured droplet sizes range over four orders of magnitude, from 0.1 to 1000 μm.

**Fig. 4:**
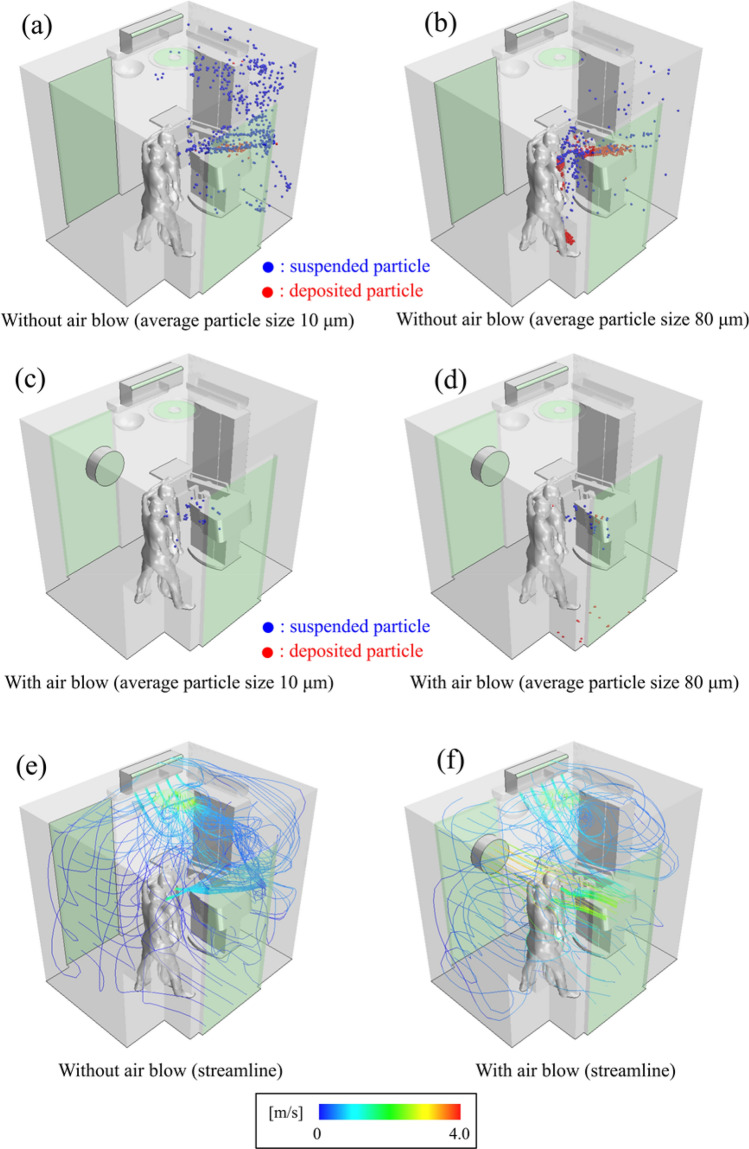
Snapshots of the simulations

Figure 4 shows simulation data of an MMG laboratory with an examiner and a patient with two doors open, with air conditioner, ventilation fan on, and with/without blower fan on.

Figure 4a and b show snapshots of a number of simulations, where the aerosols exhaled by the examiner and patient with two doors open, with air conditioner, ventilation fan, and with blower fan on, are visualized. When aerosol dynamics are considered, the smaller aerosol fraction, which was exhaled by the examiner and patient, drifts mostly in the room, but some are evacuated from the ceiling fan (Fig. 4a). The larger aerosol fraction exhibits the same behavior, except that some adhere to the survey instrument or floor (Fig. 4b). It should be noted that the two doors are open and located diagonally from each other in the room. Opening the door is generally considered best for ventilation; however, this simulation indicates that only having the door open will not be sufficient ventilation for removing an aerosol.

Figure 4c and d both display forced ventilation with a blower from one door to the other. About 97.9% of the smaller fraction of the aerosols is removed by forced airflow (Fig. 4c, Table 1). Forced airflow removed the smaller and larger fractions of aerosols by about 97.9% and 97%, respectively. It should be noted that forced airflow dramatically reduced the adhered large particles from 76.1% to 1.4% with doors open (Fig. 4c, d, Table 1).

**Table 1:** Particle emission rate with/without air blow

	All particles	Floating	Exhale	Adhere
Large particles	Without air blow			
Avr. 80 μm	1500	264	95	1141
%	100	17.6	6.3	76.1
Small particles	Without air blow			
Avr. 10 μm	1500	676	749	75
%	100	45	50	5
Large particles	With air blow			
Avr. 80 μm	1500	26	1452	22
%	100	1.6	97	1.4
Small particles	Without air blow			
Avr. 10 μm	1500	32	1468	0
%	100	2.1	97.9	0

The streamline simulation showed that the turbulence did not occur in this setting/scenario (Fig. 4). To remove the aerosol from the room, the examiner and the patient should be inside the path of the jet flow from the blower fan. This simulation was performed on tall patients, who was 175 cm heights, with similar results. (Suppl. Figure 1, Suppl. Table 1).

**Fig. 5:**
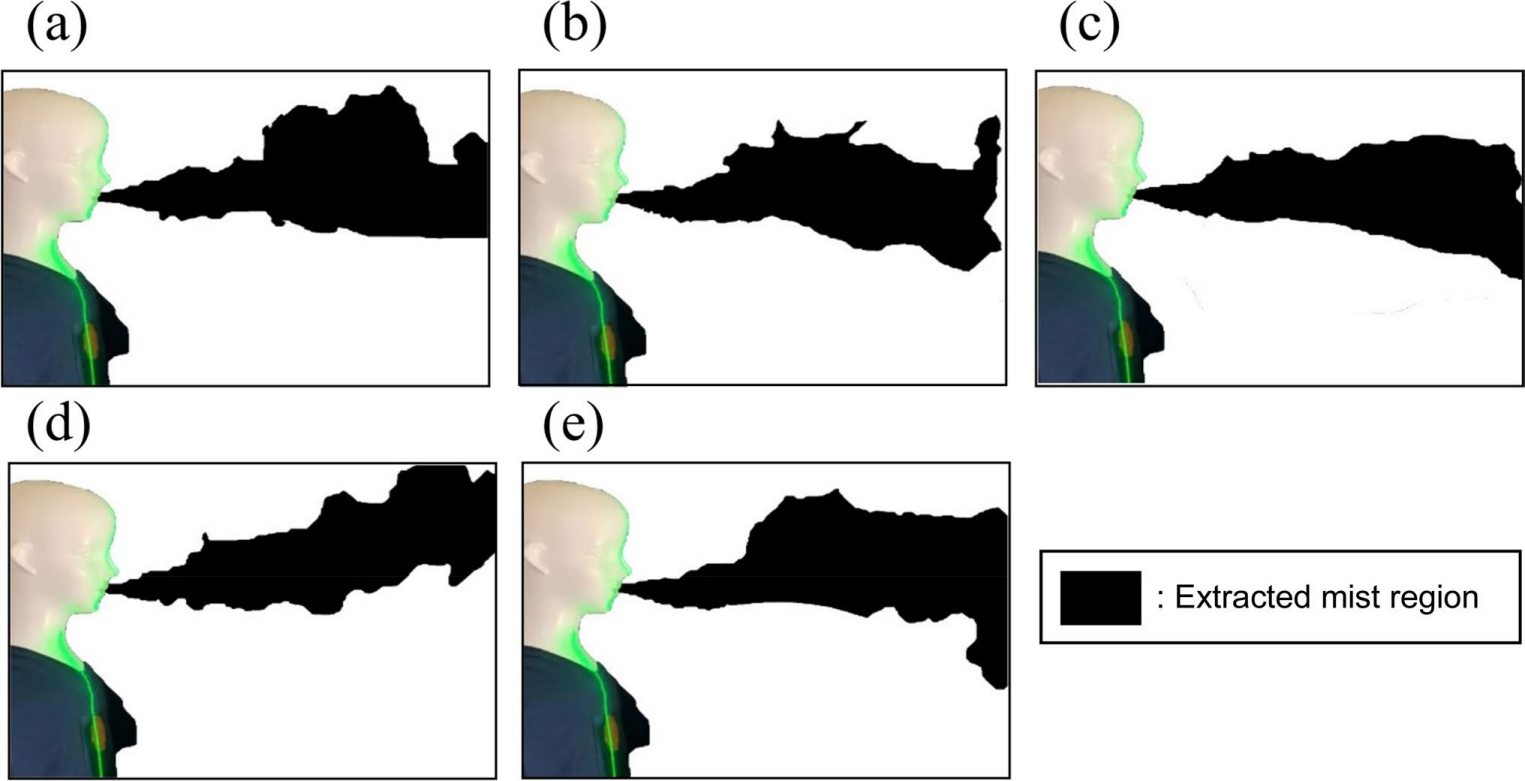
Time series change of mist region in without air blow condition (**a** 11.0 s, **b** 12.0 s, c 13.0 s, **d** 14.0 s, **e** 15.0 s)

**Fig. 6:**
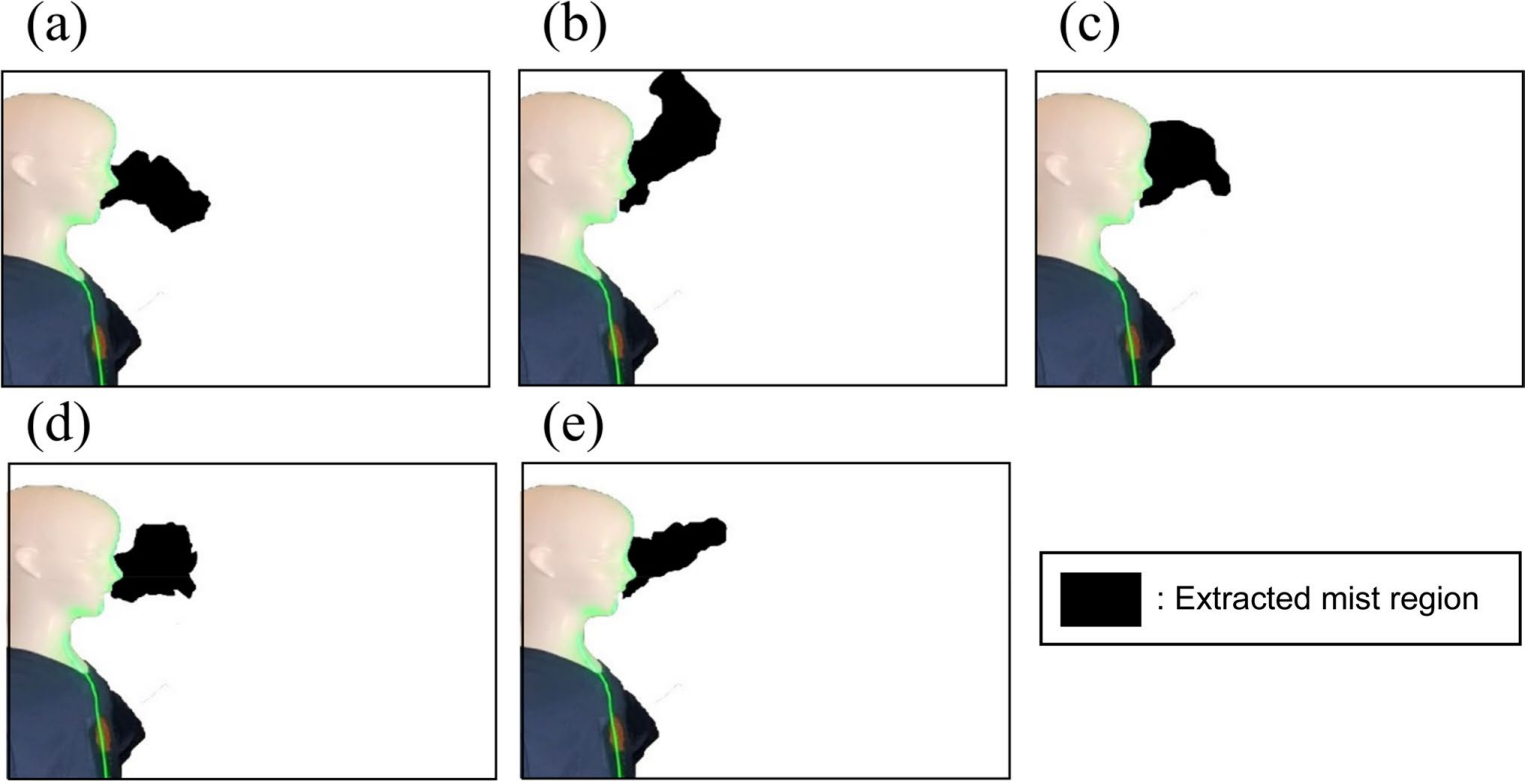
Time series change of mist region in with air blow condition (**a** 11.0 s, **b** 12.0 s, c 13.0 s, **d** 14.0 s, **e** 15.0 s)

The mist aerosol verification experiment was performed in the mobile MMG laboratory room in ‘Cosmos’ to verify the validity of our simulation study (Fig. 5). The mist aerosol was immobile and floating in the room without air blow. With the gentle breeze air blow setting by the blower fan, most aerosols were eliminated from the room. The results of this experiment are in good concordance with our computational model. We quantified the scattered area of the mist aerosol in this verification experiment. The forced wind significantly reduced the mist aerosol scattering area from 85 to 92% at any given time. (Fig. 6).

## Discussion

SARS-CoV-2 may have the potential to be transmitted through aerosols. Therefore, room ventilation can effectively limit the concentration of SARS-CoV-2 RNA in aerosols [20]. However, there is not enough evidence of specific measures related to ventilation in a clinical setting/scenario. Efficient removal of an aerosol or droplet exhaled from an examiner and patient in a laboratory room would prevent airborne transmission of COVID-19 in clinical situations. Previously, the importance of ventilation was emphasized [20], but no concrete method was stated. Viruses may be transmitted by big aerosols or droplets that fall on surfaces as well as floating small droplets. Therefore, we should recognize specific areas within the laboratory where drops or aerosols may frequently adhere to and thereby need to be wiped down for the removal of the attached virus.

The clinical setting/scenario is often difficult to ventilate from the point of view of privacy, temperature, or exposure protection, especially the examination room in the mobile MMG bus, which is too small in size to place a large additional ventilation machine. It is known that some personal protection measures for the general public, such as the wearing of masks, avoidance of busy crowds, and effective sanitization of high-risk areas in the hospital, may reduce the risk of exposure to the airborne virus [3], decrease the transmission of airborne SARS-CoV-2, and thereby protect the medical staff [5]. Cancer screening is an area in the hospital that should have a high priority for reducing the risk of COVID-19 infection, to extend or manage the healthy life expectancy of patients. Our study can address any of the concerns presented above using a clear and simple approach.

The results of our simulation and verification study indicate important and concrete evidence supporting the prevention of infection in patients and medical staff during radiographic examination. The simulation results indicate that the absence of external airflow limited the effect of ventilation; moreover, the air conditioner created turbulence, which caused pneumatic agitation of aerosols in the clinical scenario.

This study analyzes “airflow” and “movement of particles under gravitational force due to airflow” at the same time based on actual measurements. Even if the “airflow” is the same, the motion differs between small and large particles because the Stokes numbers of small and large particles are different, about 0.01 and 0.1, respectively; most light particles following along with the airflow path, but heavy particles do not follow and settle, so large particles are deposited (Fig. 4b). A streamline is a line that connects the velocity vectors of the “airflow” itself. From the above discussion of Stokes number, the streamline is considered to be close to the trajectory of light particles, but different from the trajectory of large particles (Fig. 4e, f).

The flow from the air conditioner pushes the flow from the person’s mouth from the front wall along to the back wall, preventing the exhaled air from being expelled quickly from the ceiling fan (Fig. 4e). Even if the particles are light enough, they are not discharged much from the ceiling ventilation fan in Fig. 4a and b; the particles wrap around the back wall instead of going to the ceiling ventilation fan. This is because of the flow from the air conditioner, which is essential in clinical practice.

A high-speed jet is generated from the blower and reaches the front door. The transport of particles by this jet plays an important role in the reduction of deposited particles due to the blower ventilation in Fig. 4d. For indoor ventilation that contains particles with sedimentation, such as large particles, the conventional concept of replacement ventilation of replacing air is not sufficient, and particles are captured with a flow that is an order of magnitude higher than the sedimentation rate as in this case. It is considered effective to transport the particles to the outside.

All simulations performed (e.g. Figures 4 and 5) lead to the conclusion that it is not enough just to open the door or window even if two doors are open. The most important measure is to create a gentle wind forcing from one door to the other. The forced wind blow must be a breeze because a strong wind blow produces turbulence, which causes the spread of smaller aerosol particles in the room (data not shown). Furthermore, the direction of the forced wind should be from one door to the other, and the patient and examiner should be located inside the path of the jet flow (Fig. 4).

The ventilator located in the center of the ceiling in this room discharged the air-flow from the air conditioner but did not work well for room ventilation. To have adequate aerosol ventilation, perhaps the ventilator and air supply equipment should be located diagonally from each other with the patient and examiner in the center.

In actual operation, the examiner and the patient almost always wear masks in the laboratory. Therefore, we conducted a demonstration experiment to confirm the aerosol scatter proof effect on the masked model (Suppl. Figure 3). The results indicated that the aerosol diffusion-preventing effect of the non-woven mask was insufficient because the mist aerosol leaked especially around the individual’s face even if they wear a mask. The most notable aspect with respect to the MMG examination is that the examiner cannot perform the examination without close contact with the patient. Therefore, it seems essential to take measures for aerosols even if everyone wears a mask.

Our results indicate that appropriate ventilation with gentle wind may effectively limit the aerosols in an X-ray laboratory. Further work should explore the infectivity of an aerosolized virus in such clinical scenarios.

## Supplementary Information

Below is the link to the electronic supplementary material.

Supplementary file 1 (PPTX 15805 KB)
