# Supplementary material for: Practical approach to prevent COVID-19 infection at breast cancer screening
Source: Breast Cancer. 2021 Apr 2;28(4):969–76. doi: 10.1007/s12282-021-01235-y (PMC8018689; doi:10.1007/s12282-021-01235-y)
Supplement: Supplementary file 1 — (PPTX 15805 KB) [file 12282_2021_1235_MOESM1_ESM.pptx]

## Slide 1
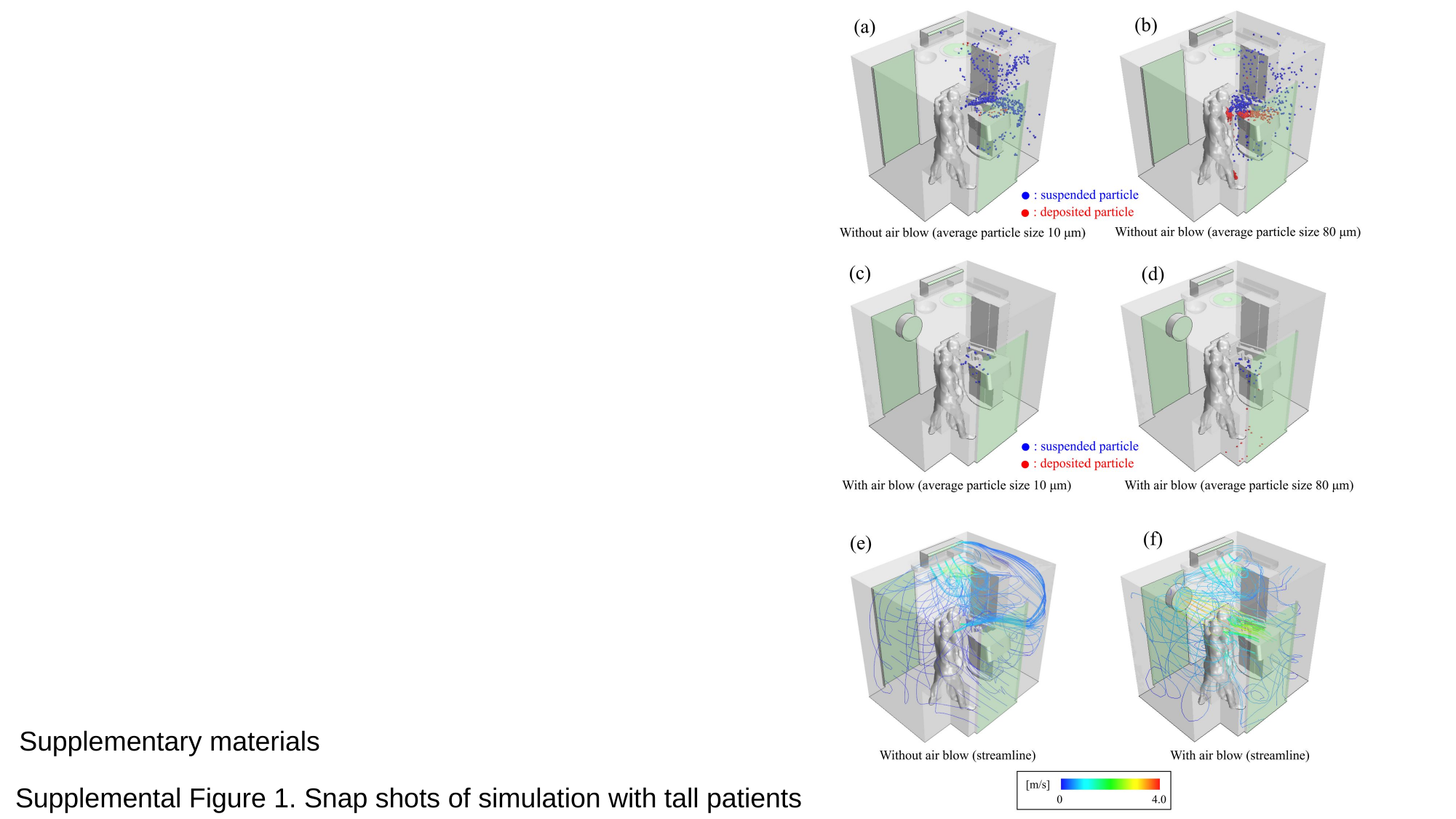

Supplementary materials
Supplemental Figure 1. Snap shots of simulation with tall patients

## Slide 2
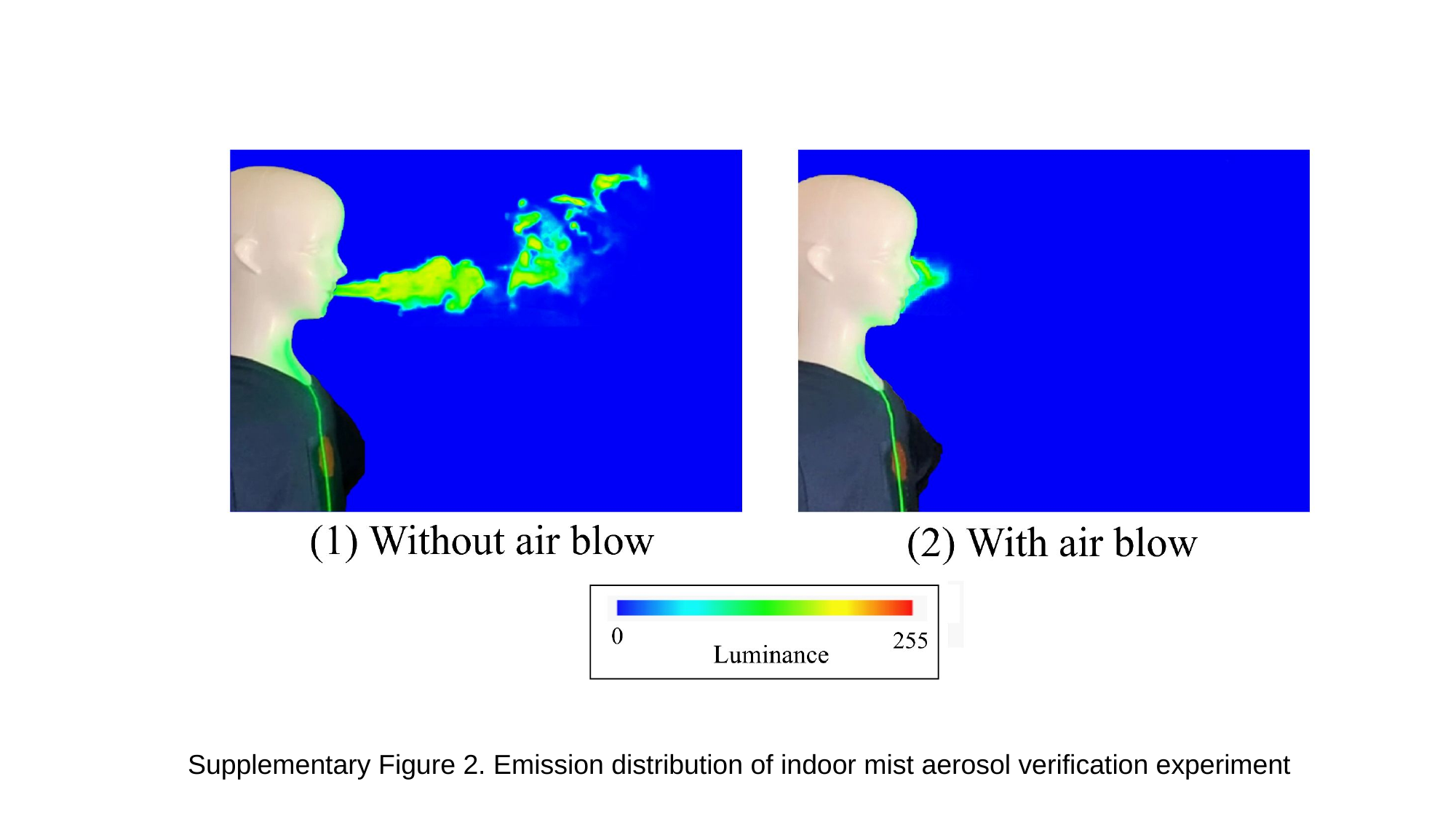

Supplementary Figure 2. Emission distribution of indoor mist aerosol verification experiment

## Slide 3
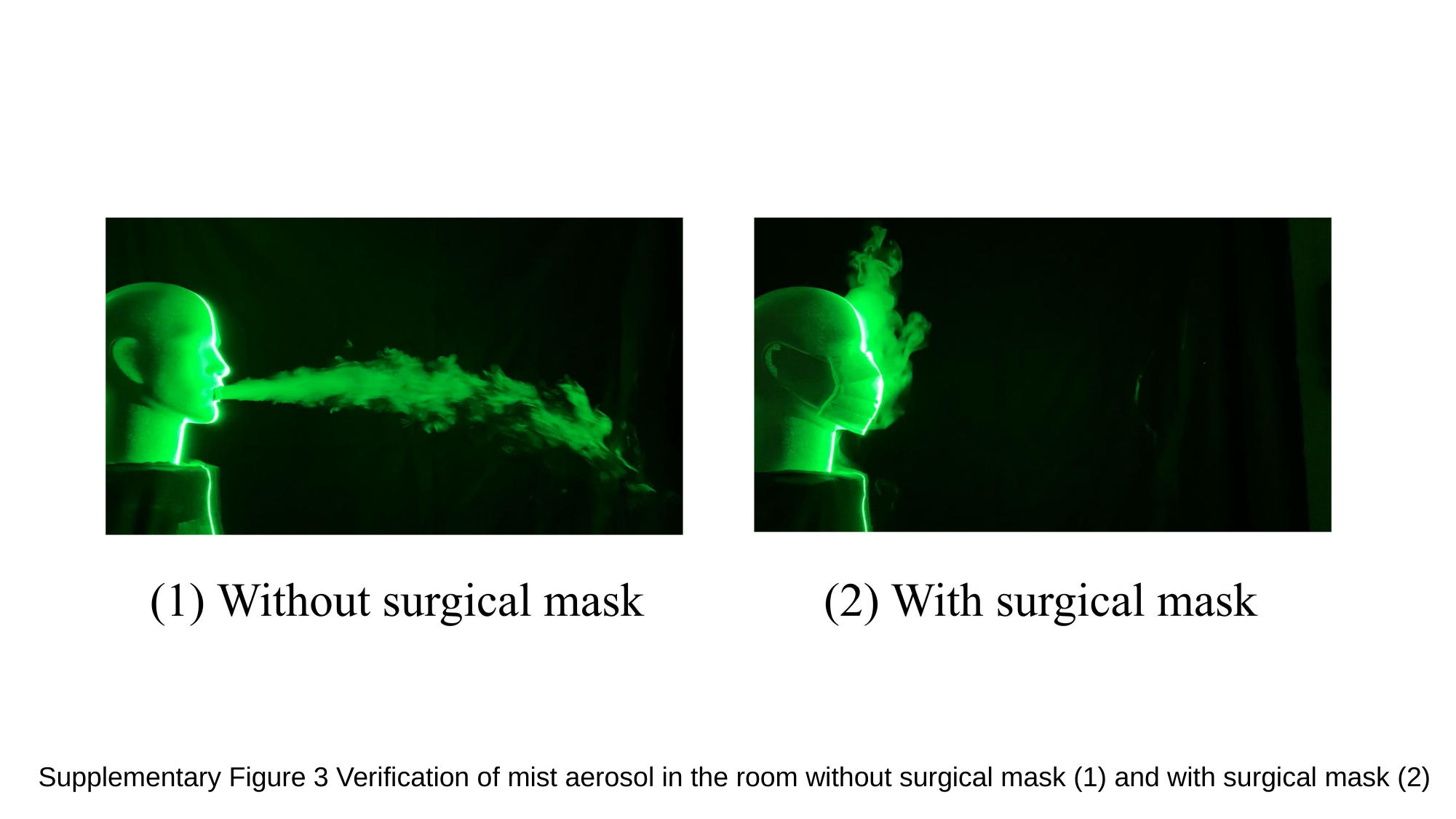

Supplementary Figure 3 Verification of mist aerosol in the room without surgical mask (1) and with surgical mask (2)

## Slide 4
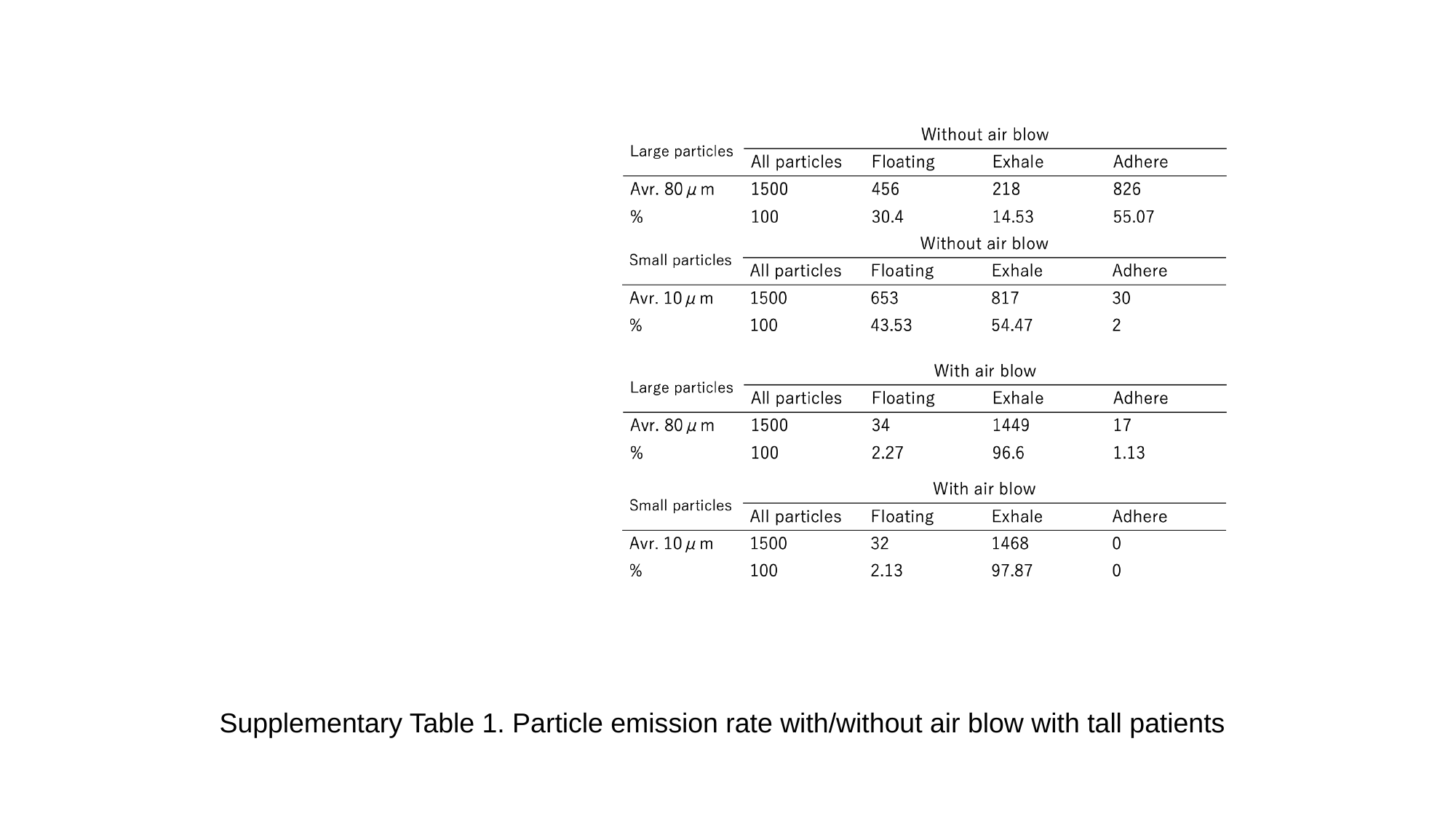

Supplementary Table 1. Particle emission rate with/without air blow with tall patients
